# Estimating Population Cause-Specific Mortality Fractions from in-Hospital Mortality: Validation of a New Method

**DOI:** 10.1371/journal.pmed.0040326

**Published:** 2007-11-20

**Authors:** Christopher J. L Murray, Alan D Lopez, Jeremy T Barofsky, Chloe Bryson-Cahn, Rafael Lozano

**Affiliations:** 1 Institute for Health Metrics and Evaluation, University of Washington, Seattle, Washington, United States of America; 2 School of Population Health, University of Queensland, Herston, Queensland, Australia; 3 Harvard Initiative for Global Health, Cambridge, Massachusetts, United States of America; 4 Director General de Información, Secretería de Salud, Mexico DF, Mexico; Umeå University, Sweden

## Abstract

**Background:**

Cause-of-death data for many developing countries are not available. Information on deaths in hospital by cause is available in many low- and middle-income countries but is not a representative sample of deaths in the population. We propose a method to estimate population cause-specific mortality fractions (CSMFs) using data already collected in many middle-income and some low-income developing nations, yet rarely used: in-hospital death records.

**Methods and Findings:**

For a given cause of death, a community's hospital deaths are equal to total community deaths multiplied by the proportion of deaths occurring in hospital. If we can estimate the proportion dying in hospital, we can estimate the proportion dying in the population using deaths in hospital. We propose to estimate the proportion of deaths for an age, sex, and cause group that die in hospital from the subset of the population where vital registration systems function or from another population. We evaluated our method using nearly complete vital registration (VR) data from Mexico 1998–2005, which records whether a death occurred in a hospital. In this validation test, we used 45 disease categories. We validated our method in two ways: nationally and between communities. First, we investigated how the method's accuracy changes as we decrease the amount of Mexican VR used to estimate the proportion of each age, sex, and cause group dying in hospital. Decreasing VR data used for this first step from 100% to 9% produces only a 12% maximum relative error between estimated and true CSMFs. Even if Mexico collected full VR information only in its capital city with 9% of its population, our estimation method would produce an average relative error in CSMFs across the 45 causes of just over 10%. Second, we used VR data for the capital zone (Distrito Federal and Estado de Mexico) and estimated CSMFs for the three lowest-development states. Our estimation method gave an average relative error of 20%, 23%, and 31% for Guerrero, Chiapas, and Oaxaca, respectively.

**Conclusions:**

Where accurate International Classification of Diseases (ICD)-coded cause-of-death data are available for deaths in hospital and for VR covering a subset of the population, we demonstrated that population CSMFs can be estimated with low average error. In addition, we showed in the case of Mexico that this method can substantially reduce error from biased hospital data, even when applied to areas with widely different levels of development. For countries with ICD-coded deaths in hospital, this method potentially allows the use of existing data to inform health policy.

## Abstract 2

Working in Mexico and using vital registration data, Chris Murray and colleagues achieved encouraging results with a new method to estimate population cause-specific mortality fractions.

## Editors' Summary

**Background.:**

Governments and international health agencies need accurate information on the leading causes of death in different populations to help them develop and monitor effective health policies and programs. It is pointless investing money in screening programs for a type of cancer in a country where that cancer is very rare, for example, or setting up treatment centers for an infectious disease in a region where the disease no longer occurs. In developed countries, most deaths are recorded in vital registration (VR) systems. These databases record the specific cause of death, which is assigned by doctors using the International Classification of Diseases (ICD), an internationally agreed-upon list of codes for hundreds of diseases. Across the developing world, however, only one death in four is recorded by VR systems; in some very poor countries, only one death in 20 is recorded accurately. With this paucity of cause-of-death data, developing countries cannot make good decisions about how to spend their limited resources.

**Why Was This Study Done?:**

The establishment of full VR systems in all developing countries will take time and may not always be possible, but many of these nations already collect ICD-coded data on in-hospital deaths. Unfortunately, this information does not accurately reflect the causes of death across whole populations. For example, the diseases that affect rich people differ from those that affect poor people, and rich people are more likely to die in hospital than poor people. Thus, although for each cause of death, the number of deaths in hospital equals the total number of deaths in the community multiplied by the proportion of deaths occurring in hospital, this proportion is different for each cause. If these proportions could be estimated, then in-hospital death records could be used to determine the fraction of the population that dies from each cause—the population's “cause-specific mortality fractions” (CSMFs). In this study, the researchers have devised a method that allows them to do this, and have used near-complete VR data collected between 1998 and 2005 in Mexico to test their method.

**What Did the Researchers Do and Find?:**

The researchers developed a mathematical method that estimates the proportion of deaths occurring in hospitals for people grouped together by their age, sex, and cause of death (an “age–sex–cause group”) using VR data from a subset of the whole population. They tested their method for 45 nonoverlapping but all-encompassing diseases using the Mexican VR data (which records when a person has died in the hospital). They found that if they decreased the amount of VR data used to estimate the proportion of each age, sex, cause group dying in hospital from 100% to 9%, the maximum relative error between the true and estimated CSMFs was only 12%. When they just used the VR information from the capital city (9% of the population), the average relative error in CSMFs (a measure of how much the estimated and true CSMFs differ) across all 45 causes of death was only 10%. Finally, when they used VR data for the main urban area of Mexico (where access to hospitals is good) to estimate CSMFs for the three least developed states of Mexico, the average relative errors were 20%, 23%, and 31%.

**What Do These Findings Mean?:**

These findings indicate that the researchers' method can provide accurate estimates of population CSMFs using ICD-coded cause-of-death data from deaths in hospital and VR data that cover part of the population. Even when the VR data from a developed area are used to calculate the CSMFs in a poorly developed area, the method produces a more accurate estimate than in-hospital death data used alone. Because the researchers have only tested their method for one country, additional “validation studies” need to be done using data from other countries with a good-quality VR system. If the method does work in these other settings, then existing data on in-hospital deaths could be used to determine the leading causes of death in countries with poor VR systems. Such information would be invaluable in establishing effective health policies.

**Additional Information.:**

Please access these Web sites via the online version of this summary at http://dx.doi.org/10.1371/journal.pmed.0040326.

• An accompanying paper by the same authors describes an alternative approach to collecting accurate cause-of-death data in developing countries

• The World Health Organization provides information on health statistics and health information systems, on the International Classification of Diseases, and on the Health Metrics Network, a global collaboration focused on improving sources of vital statistics and cause-of-death data

• Grand Challenges in Global Health provides information on research into better ways for developing countries to measure their health status

## Introduction

Reliable information on the leading causes of death in populations and how this death structure changes is a key element of the evidence base to guide health policies and programs. The relative speed of mortality decline for major diseases and injuries, or even reversals of mortality decline such as that observed in Africa [1–3], Eastern Europe [4,5], and parts of Asia [6], are more competently addressed in terms of public policy responses with reliable information on cause-of-death trends. Yet, despite the critical importance of population data for causes of death, availability of such data in many countries is limited. For the developing world as a whole, only about 25% of deaths are recorded by vital registration (VR) systems; in the poorest countries, this figure is closer to 5%–10% [7,8]. Recognition of the importance of good cause-of-death data for public health monitoring, even in the poorest countries, has led to increased interest in verbal autopsy (VA) as a tool for measuring population cause-specific mortality fractions (CSMFs) [9–12]. While this increased focus on VA methods and applications is welcome, current knowledge and practices need to be strengthened to improve scientific validity. For example, we have proposed a new method for assigning causes of death using VA data that does not depend on physician review and provides more accurate population CSMFs, as well as individual cause of death assignments [13]. Even if standardized instruments for VA were to be widely adopted, and methodological improvements systematically applied to enhance comparability of results, population VA data would still require new data collection through either household surveys or demographic sample surveillance systems.

Many poor nations already collect data that in fact can be used to measure population CSMFs, namely in-hospital deaths where the underlying cause of death has been coded according to the International Classification of Diseases (ICD). For example, publications from ministries of health and research studies report on mortality in hospital coded by the ICD in at least 18 sub-Saharan African countries [14–31]; essentially all countries in Latin America and many in the Caribbean also report in-hospital deaths [32–34]. While these data are extensively collected in poor countries, they are rarely used for population cause-of-death monitoring since they are likely to be highly biased. Deaths in hospital are not a random sample of deaths in the community. Some categories of individuals such as the rich or more educated are more likely to die in hospital, and causes of death among the rich are likely to be different than among the poor. Furthermore, deaths that occur in hospital are likely to be affected by the natural history of disease or injury. Motor vehicle accident deaths are less likely to occur in hospital than obstructed labor deaths, for example. The probability of dying in hospital is also likely to be a function of physical, financial, and cultural access to hospital services.

Previous studies have shown that in low-income settings VR systems capture only a small fraction of deaths in the community [8,35–38]. Arudo et al. [39] compared vital registration causes of death in rural Kenya with the results of VA in children and found substantial differences in causes of death. Whiting et al. [40] found that causes of death in hospital were similar to those detected using VA for the population over age 5 y. These prior attempts to use VR and/or hospital death data have not attempted to address the problem that deaths in hospital are not a representative sample of all deaths.

In this paper, we propose a method to obtain relatively accurate CSMFs for the population using CSMFs from in-hospital deaths. The basis of the method is the use of observed proportions of in-hospital death by age–sex–cause group to correct observed hospital CSMFs, yielding robust estimates of population CSMFs. To validate our method, we used vital registration data from Mexico for the years 1998–2005. We also explored the extent to which applying probabilities of in-hospital death in one population could be used to estimate population CSMFs in another.

## Methods

### The Model and Definitions

We begin with the following definitions:

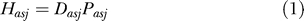
where *H_asj_* is the number of deaths in hospital for age group *a*, sex *s* from cause *j*, *D_asj_* is the number of population deaths in age group *a*, sex *s* from cause *j*, and *P_asj_* is the proportion of deaths in age group *a*, sex *s* from cause *j* that occur in hospital. In addition, the population cause-specific mortality fraction is simply the number of deaths from cause *j* divided by all deaths:

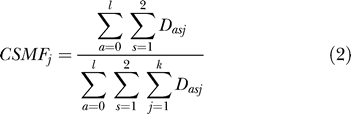



It follows from Equation 1 that we can estimate deaths from cause *j* in an age–sex group by dividing hospital deaths by the proportion of deaths that are expected to occur in hospital:

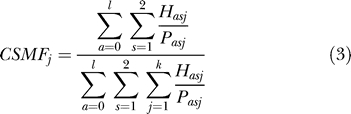



If we are able to estimate the values of *P_asj_* for a population, then in-hospital deaths can be easily corrected to yield population CSMFs.

To operationalize Equation 3, we require two information sources: (a) deaths in hospital by age and sex accurately assigned an underlying cause of death according to the ICD [41], and (b) an estimate of the proportion of in-hospital death by age, sex, and cause group, values of *P_asj_* obtained from a subset of that population or a similar population in another country. Nearly all middle-income and many low-income countries record in-hospital deaths by cause, and in a number of them the cause attribution may be of sufficiently high quality to obtain more detailed data that would allow tabulation by age, sex, and cause. The challenge for operationalizing this method is to obtain a reasonable estimate of *P_asj_*. Estimates of *P_asj_* can only be obtained where complete or near-complete VR systems are available that accurately assign the underlying cause of death and whether the death occurred in hospital. In countries that have complete VR systems for the whole country, estimating CSMFs using Equation 3 is not necessary, because the VR system will directly yield population CSMFs. For countries without complete VR systems, however, there may be a functioning VR system for a particular subset of the population such as urban areas, or selected states and provinces. These partial VR data can be used to estimate *P_asj_* values if the death certificate also includes information on whether the death occurred in hospital. In this way our method allows estimation of population CSMFs from in-hospital deaths. The strength of this approach depends on the accuracy of *P_asj_* estimates for a subset of the population or some other community. This accuracy in turn depends on how stable *P_asj_* values are across communities with different socioeconomic levels and over time. In this study, we use complete VR data for Mexico, which includes whether a death occurred in hospital, to test whether various approaches to estimating *P_asj_* values based on partial VR data provide robust estimates of CSMFs.

### Validation

We validated this approach using individual death records from Mexico 1998–2005 (see Table 1). Vital registration is estimated to be over 90% complete in Mexico and closer to 95% complete for adults [8]. Mexico collects information on the location of death (in-hospital or not), so that we can use in-hospital deaths in Mexico to assess whether our predicted population CSMFs are close to the observed population CSMFs from the vital registration data. Of the more than 450,000 deaths in Mexico in 2005, 44% occurred in hospital and 56% out of the hospital. Overall, 97% of deaths were certified by a doctor and of those, 29% were certified by the attending doctor, 14% by a forensic scientist, and 57% by another doctor. Of those deaths occurring outside the hospital, 30% were certified by the attending physician, 19% by a forensic scientist, 47% by other doctors, and 4% by non-doctors.

**Table 1:**
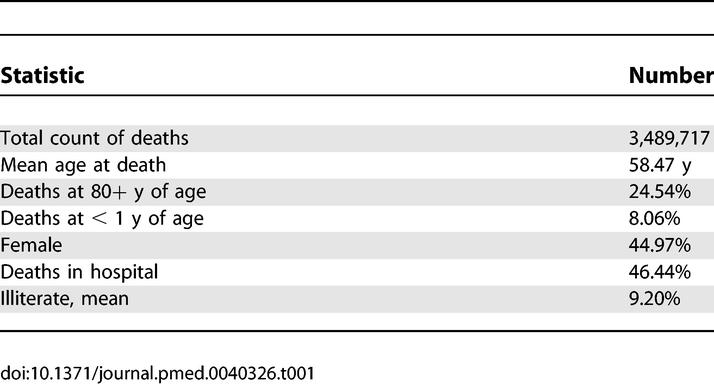
Descriptive Statistics for Vital Registration Death Data in Mexico 1998–2005

Mexico's states also represent a tremendous range of socioeconomic and health conditions. For example, the states of Nuevo Leon and the Federal District have higher purchasing power parity-adjusted per capita GDP than Portugal or Greece, whereas the poorest Mexican states like Oaxaca and Chiapas have PPP-adjusted per capita GDP lower than Swaziland and Cape Verde [42,43]. Infant mortality ranges from almost 30 per 1,000 live births in Chiapas and Oaxaca—higher than Cape Verde—to around 14 in states such as Nuevo Leon and Distrito Federal, rates close to Bulgaria and Romania [44,45]. Moreover, the Mexican state of Guerrero has a higher maternal mortality ratio (MMR) than Botswana, whereas the Mexican state of Colima has a lower MMR than France [46,47]. Mexico may therefore be used to test the applicability of this approach for a wide range of developing countries.

We based our analysis on 45 cause groups that are mutually exclusive and collectively exhaustive. To determine these, we started with the Global Burden of Disease cause list adjusted to the U.S. cause-of-death profile, which includes 109 causes [48]. The GBD cause list maps the entire detailed three- and four-digit ICD codes into a more manageable set of cause clusters that are relevant to public health decision-making; this cause list is used by WHO for annual reporting of causes of death [49]. To avoid including small causes of death that might be subject to large sampling errors, we included only causes that account for more than 0.5% of deaths, except for a composite category “other communicable and maternal conditions” which represents 0.41% of all deaths. All other deaths were grouped into broad-cause residual categories which preserve the basic Global Burden of Disease (GBD) cause structure and yield 45 cause groups (see Table 2, ordered by percent of total deaths, and Table S1). Compared to other published VA validation studies, this is a very detailed set of causes to evaluate [11,12].

**Table 2:**
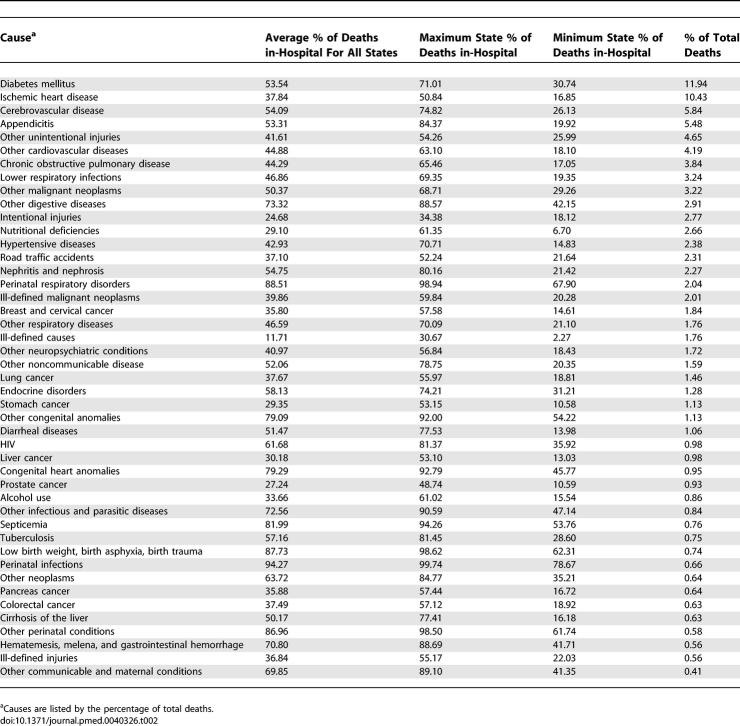
The Average, Minimum, and Maximum Fraction of in-Hospital Deaths for All 45 Causes in All States in Mexico 1998–2005

Our primary measure of method validity is the average relative error (ARE) for the 45 CSMFs. This metric can be calculated for any population for which CSMFs are being predicted. Formally, it is defined as:

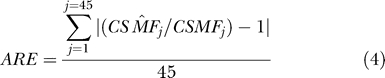
where CSMF*_j_* is the observed population CSMF for cause *j*, and 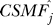 is the predicted CSMF. This metric directly measures the deviation between estimated and true CSMFs. Sensitivity and specificity for an individual cause of death cannot be measured, as this method only generates population CSMFs.

We tested this approach in two ways. First, we demonstrated that the method can provide good estimates of population CSMFs using a range of hypothetical coverage of national vital registration data. The values of *P_asj_* for a country can be estimated using the available VR data in a country. We simulated partial VR coverage in Mexico by using VR data from the most socioeconomically advanced states and progressively adding lower socioeconomic-status states. We ordered states on the basis of the literacy rate from the 2000 Census. We have tested alternative ways of ordering states such as by income, and these changes have not had a qualitative effect on the results. In other words, we assumed that most VR data come from the more developed parts of the country, especially in nations with low levels of VR coverage. For each level of partial VR coverage, we computed new *P_asj_* estimates and used this set of probabilities to correct Mexico's hospital CSMFs to estimate population CSMFs. Even based on an analysis of nearly 3.5 million deaths, we examined 45 causes-of-death by 20 age groups, by sex, so that some of these groups have small numbers. Consequently, we set the probability of in-hospital death to zero for those groups with fewer than three total deaths.

Second, we explored whether *P_asj_* values measured in one population can be used to estimate population CSMFs using in-hospital deaths in another community. We used VR data for 1998–2005 for the Distrito Federal and the Estado de Mexico, which together form the main urban and periurban center in Mexico, to calculate *P_asj_* values. We would expect that an urban area such as these two together would have higher access to hospital services than a poor rural area. We then applied these fractions of in-hospital deaths to the three poorest states in Mexico: Oaxaca, Chiapas, and Guerrero. The difference between the capital city area and these states in terms of income, educational attainment, and access to hospital services is quite marked, making this a strong test of the generalizability of the method to other countries where values of *P_asj_* would need to be estimated from data in a neighboring country.

## Results

**Figure 1:**
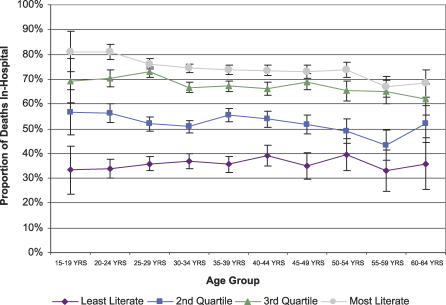
Proportion of HIV/AIDS Deaths That Were in Hospitals, versus Age Group, According to Community Literacy Quartiles, Mexico 1998–2005 Data are means and 95% confidence intervals.

**Figure 2:**
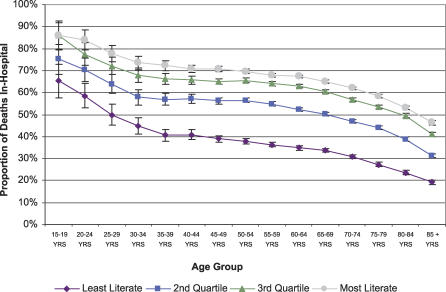
Proportion of Diabetes Deaths That Were in Hospitals, versus Age Group, According to Community Literacy Quartiles, Mexico 1998–2005 Data are means and 95% confidence intervals.

**Figure 3:**
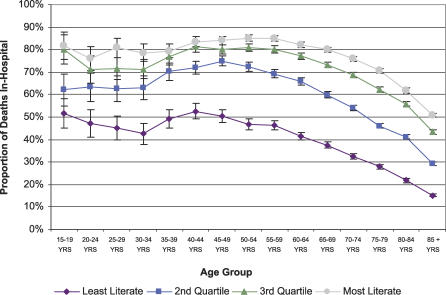
Proportion of Cerebrovascular Disease Deaths That Were in Hospitals, versus Age Group, According to Community Literacy Quartiles, Mexico 1998–2005 Data are means and 95% confidence intervals.

**Figure 4:**
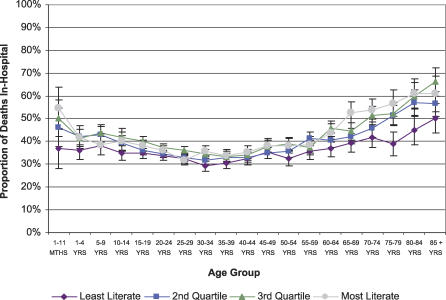
Proportion of Road Traffic Accident Deaths That Were in Hospitals, versus Age Group, According to Community Literacy Quartiles, Mexico 1998–2005 Data are means and 95% confidence intervals.

Table 2 summarizes the average, minimum, and maximum proportion of in-hospital deaths for all 45 causes in all 32 states in Mexico. Perinatal infections represent the cause of death with the highest average proportion of deaths that occur within a hospital at 94%, while the average proportion for the intentional-injuries group is the lowest at 25%. Across states and causes, the proportion of in-hospital deaths varies from 7% for nutritional deficiencies to 100% for perinatal infections. Figures 1–4 show how the in-hospital fraction of death differs by age group and cause. The figures also show the fractions dying in hospital for four subgroups of the Mexican population created by dividing the population into four groups on the basis of population literacy. These subgroups serve to demonstrate how socioeconomic status affects the overall probability of dying in hospital. For HIV/AIDS, diabetes mellitus, and cerebrovascular disease, the proportion dying in hospital at any age group is lower in municipalities with lower socioeconomic status as indicated by literacy rates. For road traffic accidents, however, there is no marked difference by literacy status in the proportion of in-hospital deaths, as might be expected. Diabetes and cerebrovascular diseases show a generally declining proportion of deaths in hospital as a function of age. There is a weaker trend by age for HIV/AIDS and the proportion dying in hospital increases slightly for road traffic accidents. These four causes illustrate that the proportion of in-hospital deaths is a distinct function of age, cause, and level of community development. This diverse pattern confirms that CSMFs based solely on in-hospital deaths are likely to be inaccurate.

**Figure 5:**
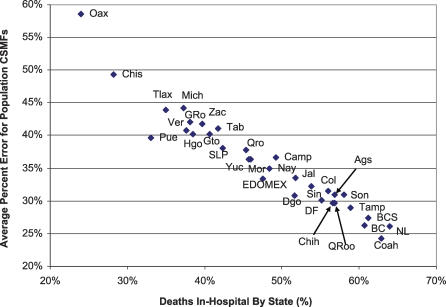
Average Relative Error in Population CSMFs when Based on Hospital CSMFs by State versus the Proportion of All Deaths Occurring in-Hospital, Mexico 1998–2005 Each point represents a state in Mexico. Legend: Ags, Aguascalientes; BC, Baja California; BCS, Baja California Sur; Camp, Campeche; Chih, Chihuahua; Chis, Chiapas; Coah, Coahuila de Zaragoza; Col, Colima; DF, Distrito Federal; Dgo, Durango; EDOMEX, Estado de Mexico; GRo, Guerrero; Gto, Guanajuato; Hgo, Hidalgo; Jal, Jalisco; Mich, Michoacan de Ocampo; Mor, Morelos; Nay, Nayarit; NL, Nuevo Leon;Oax, Oaxaca; Pue, Puebla; Qro, Queretaro de Arteaga; QRoo, Quintana Roo; Sin, Sinaloa; SLP, San Luis Potosi; Son, Sonora; Tab, Tabasco; Tamp, Tamaulipas; Tlax, Tlaxcala; Ver, Veracruz-Llave; Yuc, Yucatan; Zac, Zacatecas.

Indeed, Figure 5 demonstrates the inaccurate nature of hospital CSMFs when used without correction to estimate population CSMFs. The figure shows average relative error for hospital CSMFs as a function of the percentage of deaths in-hospital for each Mexican state. As expected, the average percent error steadily rises as the proportion of deaths in hospital falls. In other words, in states with a smaller proportion of in-hospital deaths, the effects of selection bias on the hospital CSMFs are greatest. Specifically, average error ranges from 25% in the most developed states in the north of Mexico to 50% to 60% in the least-developed communities of Chiapas and Oaxaca where the proportion of deaths that occur in hospitals is less than 30%. The data in Figure 5 provide strong empirical validation for the hypothesis that, without correction, hospital CSMFs in poor communities are highly inaccurate. The relationship between error and percent of in-hospital deaths appears to be nearly linear.

**Figure 6:**
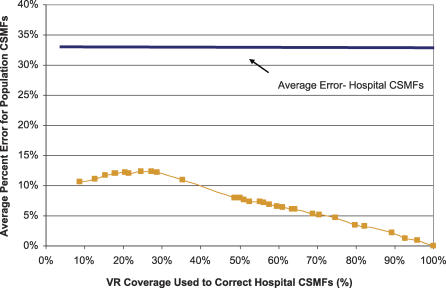
Population CSMFs Average Relative Error for 45 Cause Groups ARE was calculated as a function of the percentage of Mexican VR data used to assess the proportion of deaths in each age, sex, and cause group dying in hospital.

Figure 6 systematically explores the relationship between the amount of VR data used to calculate the *P_asj_* values in Mexico (from 9% to 100%) and the average relative error across 45 causes of death at the national level. When 100% of VR data is used, by definition, the *P_asj_* values are correct and ARE is zero. As the proportion of deaths used to estimate the *P_asj_* values drops, ARE increases. Nevertheless, ARE based on 9% to 35% of all deaths captured in the VR system ranges from only 10% to a maximum of 12%. For comparison, Figure 6 also shows ARE if in-hospital deaths were used to estimate population CSMFs without correction, at the national level, namely 33%. The conclusion emerging from Figure 6 is that even if VR in Mexico covered only a small fraction of the country's most developed states, our methods suggest that we would be able to measure CSMFs quite accurately if data on causes of death in hospital were available. We hypothesize that ARE remains relatively stable when we estimate population CSMFs using between 9% and 35% of Mexico's VR data because the better-off states may have a similar hospitalization pattern, so that adding further data does not substantially affect *P_asj_*. Using only 9% of deaths (from Distrito Federal), Table 3 compares the average difference over states between true and predicted CSMFs, where predicted CSMFs are calculated from hospital data or using the method described in Equation 3. The average difference is closer to zero for most, but not all causes of death; moving from −1.4% to −0.79% for diabetes and from 0.55% to −0.13% for road traffic accidents, but increasing slightly for lower respiratory disease. The interquartile range in the difference between true and predicted CSMFs using our method varies widely over states for other unintentional injuries (from −0.54% to 0.94%), but varies little for causes such as other neoplasms and gastrointestinal hemorrhage.

**Table 3:**
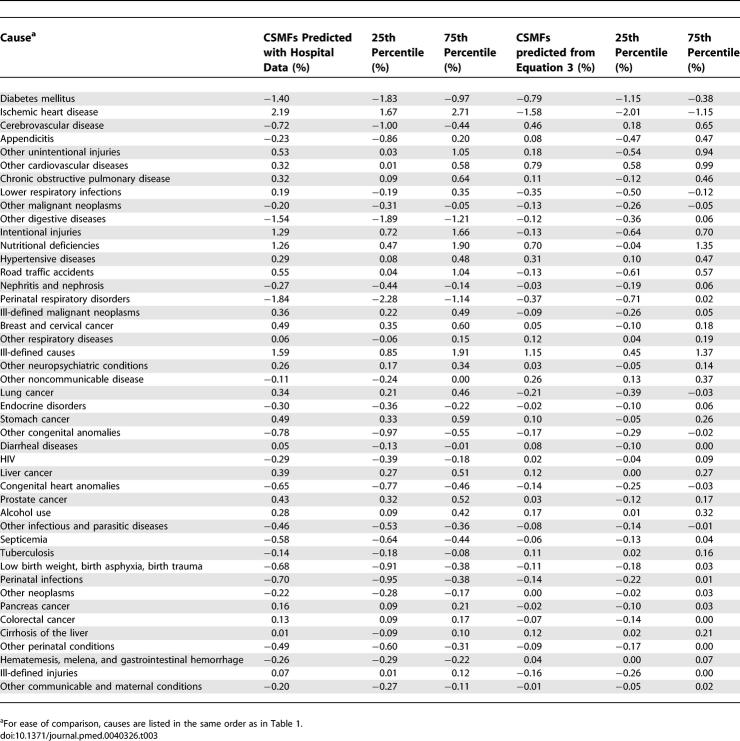
The Average, 25th, and 75th Percentile Difference over States between True and Predicted CSMFs Using Hospital Data and the Correction Method from Equation 3

**Figure 7:**
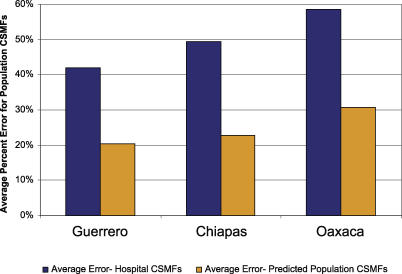
Population CSMFs Average Relative Error for the Three Least-Developed Mexican States Average relative error was estimated on the basis of the proportion of in-hospital deaths by age, sex, and cause groups observed in Distrito Federal and Estado de Mexico.

Using VR data from the capital city and surrounding communities (Distrito Federal and Estado de Mexico) to estimate the *P_asj_*, Figure 7 demonstrates what would be the ARE for the three least developed states in Mexico. In the state with the lowest fraction of deaths in hospital, Oaxaca, the ARE is 30% using our correction method. The ARE across the 45 CSMFs is even lower for the states of Guerrero and Chiapas. While these levels of error are much higher than we obtain at the national level, the results still demonstrate the possibility of estimating plausible CSMFs for a large set of causes even in settings where the *P_asj_* values cannot be measured directly, but must be borrowed from another population.

## Discussion

In this paper, we have demonstrated that when high-quality ICD-coded data on deaths in hospital and high-quality ICD-coded data from vital registration from a small subset of the population or a similar population are available, population CSMFs can be estimated with an acceptable level of error. The results are robust even when using less than 10% of Mexico's VR data to estimate the proportion of in-hospital death for each age, sex, and cause group. These results are encouraging; in VA validation studies, in the best of circumstances, for much smaller and less detailed cause groups, the average percent error has been found to be substantially higher. For example, an adult VA validation study using physician coded VA found 70% average error over 23 cause groups in China [12,13]. The average error in this analysis, with more than twice as many cause groups, is markedly smaller.

The critical question for national and global mortality analyses is whether we can generalize this approach to populations outside Mexico. Given the realities of the quality of in-hospital death coding and the availability of partial VR data, we believe there are four distinct scenarios with different applications of this approach. First, there are a number of countries, such as the Dominican Republic, Ecuador, Egypt, El Salvador, Georgia, Guatemala, Haiti, Honduras, India, Iran, China, Kazakhstan, Tunisia, Morocco, Algeria, Lebanon, Mozambique, Nicaragua, Peru, Zimbabwe, Turkey, and Senegal [8,38,50–58], where reasonably accurate ICD-coded VR data are available for a subset of the population and in-hospital death data are also available. For example, in the Indian state of Maharashtra, comparison of urban death rates from the Sample Registration Scheme and the medical certification of causes of death data suggest that about 80% of urban deaths are captured [56]. In South Africa, the vital registration system is believed to capture about 90% of deaths [2]; this dataset could perhaps serve to explore how much the values for *P_asj_* differ from Mexican municipalities at the same level of development. In a similar fashion, nearly complete vital registration data for the eastern provinces of China could be used to estimate CSMFs in the poorer provinces [58]. These are countries where the approach outlined here may be directly applicable.

Second, there are countries, such as Brazil, Colombia, Costa Rica, Thailand, or the Philippines, that have VR systems that are more than 80% complete [8], but often the poorest communities are not covered and exhibit low-quality cause-of-death assignment. In these settings, our approach may have a more limited role in helping assess the population CSMFs in selected low-income communities.

Third, there are a number of countries in sub-Saharan Africa and Asia, such as Pakistan, Bangladesh, Indonesia, and Vietnam, for which deaths in hospital are recorded and assigned causes according to the ICD, but vital registration data may not be available. In these settings, it may be worthwhile using the *P_asj_* values for a neighboring country (such as India for Pakistan and Bangladesh; or South Africa, Zimbabwe, or Mozambique for other Southern African countries) to generate an estimate of the composition of hospital mortality. Using *P_asj_* values from another country will clearly be less desirable than estimating them directly for a national population, but this analysis using deaths recorded in Mexico's capital district to estimate CSMFs in poor states demonstrates that this may still work reasonably well.

Finally, for a number of sub-Saharan African countries, deaths in hospital may be recorded, but the quality of cause-of-death attribution in hospital may be poor. In these circumstances, the method proposed here may not be productively applied. Rao et al. [58] proposed a number of criteria such as the fraction of deaths assigned to ill-defined codes to evaluate the quality of ICD coding. Whiting et al. [40] demonstrated in Tanzania that it is feasible in these settings to use physician review of the medical records maintained in hospitals to assign a more accurate underlying cause of death according to ICD principles. This is a much more time-intensive and costly effort compared to using data that are already recorded in these hospitals. Nevertheless, it may be worthwhile to invest in physician review of medical records to strengthen the quality of the ICD assignment of causes of death, especially if such data can be used to estimate population CSMFs. This approach also highlights the potential benefits of increased training for accurate cause-of-death certification in hospitals in low-resource settings.

It seems plausible that, as in Mexico, the proportion of deaths in hospital in different countries would be a systematic function of individual covariates (age and sex), cause-of-death and a set of community factors that influence physical, financial, and cultural access to hospital services. Figures 1–4 show that the proportion of in-hospital death can differ significantly by literacy level, and future work could investigate how other community attributes that relate to the use of hospital services might improve estimates of population CSMFs. We have investigated using various logistic regression models of the probability of dying in hospital as a function of a wide set of individual and community covariates. This work suggests that further research may yield important insights into the determinants of dying in hospital that may strengthen our ability to predict *P_asj_* values in various populations. It is important in this regard to recognize that, based on Equation 3, community factors that scale the *P_asj_* data for all causes in an equal manner have no effect on the accuracy of this approach. This in part explains why it is possible to use *P_asj_* values from the capital city region of Mexico, where 55% of deaths occur in hospital, to estimate the CSMFs in Oaxaca, where only 25% of deaths occur in hospital.

We have validated our method using data for only one country, Mexico. It is clearly important that future research confirm our finding that *P_asj_* values are predictable using partial data or data from other populations. Such future validation studies can only be conducted in settings where there is nearly complete vital registration with good-quality ICD coding. In these settings, investigators will require access to individual death records to measure *P_asj_* and to further investigate the determinants of dying in hospital. Such validation studies will be an important step to build confidence that the approach we propose can be applied in the various settings we have outlined. One candidate would be South Africa, which now has high levels of VR coverage and a different burden of disease than Mexico, with more cases of HIV and TB.

We have proposed a method to estimate population CSMFs using in-hospital deaths. To obtain cause-specific death rates or numbers of deaths, age-specific all-cause (total) mortality rates need to be derived from some other demographic source or method [59,60]. CSMFs would then be applied to these all-cause mortality rates to generate cause-specific death rates, the information base most relevant for public policy. It is beyond the scope of this paper to discuss the extensive literature on the methods used by demographers to generate age-specific death rates from all causes. However, we recognize that in the long run the best method to generate both age-specific death rates from all causes and accurate death rates by cause is to invest in the development of national vital registration systems. Development of methods to estimate population CSMFs, while useful, should not be taken as an excuse to ignore the strengthening of national vital registration systems.

This analysis also illustrates the potential of individual-level death data to generate critical evidence for health policy. Other studies [61,62] have illustrated how individual death files can allow insights and analyses that would otherwise be impossible. WHO should encourage countries to digitize and make available to their research communities individual death records, with appropriate steps to protect privacy [63]. Not only can these data be used to estimate a variety of population rates and proportions, but they can also permit further methodological innovation tailored to local needs.

Well-informed, flexible, evidence-based health policies are more likely to support rapid health development in poor countries than current practices based largely on anecdotal or small-scale evidence about how incidence or prevalence of major diseases is changing. Cause-of-death data have traditionally been the cornerstone of such an evidence base, yet current strategies to improve data such as vital registration or verbal autopsy are unlikely to yield adequate information for health planning in poor countries in the near future. Alternative methods are required that can be applied in conjunction with these approaches to more reliably estimate the descriptive epidemiology of populations at comparatively low cost.

Estimates of population CSMFs based on in-hospital mortality data may provide one more tool in an overall approach to develop robust cause-of-death estimates for populations without complete vital registration. The method proposed here, if more widely applicable in settings with appropriate quality ICD-coded hospital data, would allow countries to utilize data already collected on in-hospital deaths to estimate the population cause-specific mortality structure, in combination with verbal autopsy data analyzed in a standardized, comparative fashion.

## Supporting Information

Table S1 ICD-10 Codes for Cause-of-Death Categories Used in This Study (19 KB XLS)

## Abbreviations

ARE: average relative error
CSMF: cause-specific mortality fraction
ICD: International Classification of Diseases
VA: verbal autopsy
VR: vital registration
